# Detroit Medical and Library Association

**Published:** 1884-05-20

**Authors:** 


### DETROIT MEDICAL AND LIBRARY ASSOCIATION.

GASTROTOMY FOR RELIEF OF INTUSSUSCEPTION.—Dr. Carstens reported a case of a child, constipated and vomiting for six days previous to his seeing him. The child had been treated with physic and injections by other physicians, but to no purpose. He advocated operation, having diagnosed intussusception, as a long, hard swelling could be felt at the junction of the transverse and descending colon. The parents objected. Tried gas generated from tartaric acid and bicarbonate of soda, but unsuccessfully. No nourishment was retained but whisky and water, and sometimes a little gruel. Finally, under ether, an incision was made from the umbilicus down, but it was impossible to get the bowel back on account of the number and strength of the adhesions. The patient died. The doctor believed in operating right off in those cases; people were inclined to wait too long.

Dr. Babcock had successfully treated a case diagnosed as intussusception in a child, with drop doses of laudanum every five minutes till the child was thoroughly narcotized, and injections of water and milk every half hour kept up for twenty-four hours.

Dr. Inglis, referring to immediate operation, believed Dr. Carstens was on the right track if one were certain of his diagnosis.

Dr. Jennings: In some cases, spontaneous recovery has occurred with sloughing off and discharge of the invaginated portion of intestine.

DIPHTHERIA.—Dr. Hoyt reported incessant vomiting as one of the sequels in a case of diphtheria.

Dr. Jennings had had five cases of diphtheria in three weeks, four of the five invading the larynx; one in an adult with recovery.

Dr. Hoyt had recently under treatment a family where two of the children had follicular pharyngitis, and two genuine diphtheria.

Dr. Shurly: It is often difficult to distinguish between membranous sore throat and diphtheria. Clinically speaking, the diseases were identical. He thought it was the duty of the physician to give the community the benefit of the doubt.

A SPEEDILY FATAL CASE OF DIABETES.—Dr. Inglis reported a case of diabetes running a rapid course—five weeks from first complaining to death. The patient lost flesh at the rate of from one to two pounds daily. He first consulted the doctor for an intolerable thirst, supposing that he was suffering from fever. The patient five years previously had had a sunstroke, with severe headache several times since. In other respects he was always healthy, with a magnificent physique. The patient suffered no pain, and had a remarkably slow pulse. Age forty-one. No treatment seemed to particularly influence the course of the disease.

Dr. Jennings referred to a case of diabetes in a school girl which he had previously reported. The disease was treated, but with no influence over its course, with codeia, ergot, bromide of potash, etc. The patient died in eight weeks.

Dr. Shurly thought one form of the disease was from brain trouble, either the result of softening or tubercular deposit, and was looking for the verification of this opinion in some postmortem. Another form was simply dietetic, and still another malarial. We ought to regard the appearance of sugar as simply symptomatic. He would treat a case with perfect rest to the nervous system, seclusion in a dark room, ice to the head, and give large quantities of codeia or morphia. This treatment would be more rational than bran mashes.

Dr. A. F. Hoke read the following report of two cases of

IRREGULAR CONTRACTION DURING THE THIRD STAGE OF LABOR.—Cazeaux admits four distinct varieties of irregular or spasmodic contractions of the uterus: 1st, of the external orifice of the neck; 2d, that of its internal orifice; 3d, that of one or more portions of the body of the uterus; and 4th, spasmodic contractions of the whole uterus. The first is considered of little importance, as the constriction is easily broken up. The third and fourth are considered by most observers of rare occurrence, and, it is said, are usually found in the practice of beginners.

I do not design in this short paper to enter into a minute or detailed description of these different forms of irregular contractions, but simply to report two cases of the second variety which came under my personal observation.

November 17, 1883, was called by Dr. Kaiser to see Mrs. B., aged 32, multipara, who had been delivered of a male child four hours previously, by a midwife. We found, on my arrival, that she had flowed quite profusely, but that the flooding had now ceased. On making palpation, found the uterus extended slightly above the umbilicus, soft, flabby and ending in a slight constriction just above the pubis. Making a vaginal examination, I found, on following up the cord, that the hand passed readily through what seemed four or five inches of soft and flabby cervix; but, on reaching the internal os, one could readily find, what was evident from the external contour of uterus, a firm constriction at the internal os. Pushing the index finger through the constriction, I found the cord nearly separated at its junction with the placenta. Dr. K. administered chloroform to profound anæsthesia, when I broke up the stricture in the usual way, *i. e.*, by first introducing the index, then the middle finger, and so on until I was able to pass my whole hand to the fundus. The placenta was only partially separated, but the pressure of the hand in the uterus, supplemented by the external pressure of the other hand over the fundus, brought on a strong pain which separated the remainder and forced both hand and placenta from the uterus. But little blood was lost, and patient made a good recovery.

Dec. 4 was called by a midwife to see Mrs. R., multipara, who had been delivered of her fourth child three hours previously. I found, on palpation, the same condition as in case No. 1, but found, on vaginal examination, that the cord and a small portion of placenta were torn entirely away. Found same condition of long, flabby neck, and protruding through the stricture, at interval os, a small portion of placenta. Gave chloroform, dilated stricture as in preceding case, and found placenta entirely separated and about a pint of clotted blood above the stricture, which was removed. Firm contraction followed, and patient made a good recovery.

The causes of these irregular contractions, as enumerated by anthorities, are various. Among many, predisposition in the uterus itself, improper manipulation, pulling at the cord, abuse of stimulating remedies, particularly ergot, may be mentioned. In the cases reported, I have no doubt it was due to defective management of the third stage. The midwives in charge, as—from my observation—is their custom, tried to complete the labor by strong traction on the cord. Traction not made in the direction of the axis of the superior strait is not very effective in delivering a placenta, even one that is detached, but one can easily conceive how pulling at the cord in the direction of the axis of the outlet, when the placenta is in the fundus, irritates the internal os, stimulating it to contraction, while the rest of uterus remains in a state of inertia. Crede's method (excluding, of course, predisposition) would, in all probability, have prevented the accident in both cases.

As Playfair aptly says, "If placental expression were always employed, if it were the rule to effect expulsion by expression instead of traction at the cord, I feel confident these irregular contractions, of the influence of which in producing hemorrhage there can be no question, would rarely, if ever, be met with."

Dr. Carstens had had only one case of hour-glass contraction, and believed that the Doctor was right when he said it was owing to neglect in the third stage of labor. When the child is born, stick to the uterus and compress it during the pains till the placenta is expelled, making but little traction on the cord.

Dr. Flinterman believed that most cases of retained placenta depended on a contracted pelvis. One patient he knew of always had a normal labor until the child was born, and then the uterus would contract, retaining the placenta, and followed by considerable hemorrhage, requiring its immediate removal by hand.

REMOVAL OF PLACENTA.—Dr. Bonning.—In the Lying-in Hospital, in Strasburgh, it is the custom to let the placenta come away by itself. In ninety per cent. of cases it would come away in half an hour. Out of three hundred cases there was only one of post partum hemorrhage, and he had not seen there a retained placenta or hour-glass contraction.

Dr. Utter followed the method advocated by Dr. Carstens.

Dr. Warner: Usually, where the case required, introduced the hand and gave a little twist, bringing the placenta away. Had never but once had a case of hour-glass contraction.

Dr. Deuel followed the same plan.

Dr. L. H. Johnson's practice was similar to Dr. Carstens', sometimes introducing the hand as Dr. Warner did. Had no bad results from such a method.

Dr. Jamieson always made pretty firm pressure over the womb while the head was clearing the vulva.

Dr. Carstens believed that hour-glass contractions were often the result of injudicious exhibition of ergot by midwives and others.

Under the head of prevailing diseases, Dr. Jennings reported a case of cerebro-spinal meningitis, Dr. Johnson a number of cases of whooping cough, Dr. Deuel tonsillitis, and Dr. Hoke measles.

Dr. Hoyt: In cases of podalic version, as soon as he could bring the body forward, delivered the head with the forceps. He inquired the usual practice.

Dr. Carstens kept the uterus firmly compressed and delivered quickly with the forceps as soon as the child's head engaged. Dr. Hoke, in addition to pulling down on the face, pushed up on the occiput. There was not much trouble in a breech presentation.—*Medical Age.*

